# Neurotoxicity and Developmental Neurotoxicity of Copper Sulfide Nanoparticles on a Human Neuronal In-Vitro Test System

**DOI:** 10.3390/ijms25115650

**Published:** 2024-05-22

**Authors:** Michael Stern, Nandipha Botha, Karen J. Cloete, Malik Maaza, Saime Tan, Gerd Bicker

**Affiliations:** 1 Institute of Physiology and Cell Biology, University of Veterinary Medicine Hannover, Bischofsholer Damm 15/102, D-30173 Hannover, Germany; michael.stern@tiho-hannover.de (M.S.);; 2 UNESCO-UNISA Africa Chair in Nanosciences & Nanotechnology Laboratories, College of Graduate Studies, University of South Africa, Muckleneuk Ridge, P.O. Box 392, Pretoria 0003, South Africa

**Keywords:** DNT, nanoparticles, migration, neurite outgrowth, NTera-2, NT-2

## Abstract

Nanoparticles (NPs) are becoming increasingly important novel materials for many purposes, including basic research, medicine, agriculture, and engineering. Increasing human and environmental exposure to these promising compounds requires assessment of their potential health risks. While the general direct cytotoxicity of NPs is often routinely measured, more indirect possible long-term effects, such as reproductive or developmental neurotoxicity (DNT), have been studied only occasionally and, if so, mostly on non-human animal models, such as zebrafish embryos. In this present study, we employed a well-characterized human neuronal precursor cell line to test the concentration-dependent DNT of green-manufactured copper sulfide (CuS) nanoparticles on crucial early events in human brain development. CuS NPs turned out to be generally cytotoxic in the low ppm range. Using an established prediction model, we found a clear DNT potential of CuS NPs on neuronal precursor cell migration and neurite outgrowth, with IC50 values 10 times and 5 times, respectively, lower for the specific DNT endpoint than for general cytotoxicity. We conclude that, in addition to the opportunities of NPs, their risks to human health should be carefully considered.

## 1. Introduction

Nanotechnology is a fast-moving area that finds applications in diverse areas, such as material science, electronic engineering, consumer goods production, environmental protection, and medicine [1]. A recent innovation in the environmental and medical fields is the use of plant extracts in nanoparticle synthesis [2,3]. Due to the involvement of auxiliar natural substances, this so-called “green nanotechnology” aims for environmentally beneficial nanoparticles free from hazardous substances. Thus, green synthesis of nanoparticles has been the area of focused research by researchers in the last years by adopting an eco-friendly approach [2]. Moreover, green synthesis methods are said to often result in nanoparticles with well-defined sizes and shapes, leading to enhanced physicochemical properties and biological activity [4]. Using avocado seed extracts, Botha et al. 2024 [4] have manufactured biogenic copper sulfide (CuS) nanoparticles of spherical and semi-spherical shape and an average size of 77.8 nm. These nanoparticles were applied as a fertilizing agent to Pinto bean seeds, in which they triggered an effect on the seed ionome and amino acid metabolome. Elemental mapping also showed that nanoparticles might act as an antimicrobial barrier due to their accumulation in the seed coat. 

There is considerable public awareness about the toxic potential associated with nanoparticles applied to the environment, including plants [5]. The small size of nanoparticles poses the risk of permeating the protective blood–brain barrier into the nervous system. A size range of less than 100 nm implicates a greater surface-to-mass ratio than that of their bulk components. Thus, nanoparticles can show a high reactivity and cell permeability. Under natural conditions, nanoparticles may be incorporated into human organs via ingestion, skin contact, and inhalation. Due to its very limited capacity for regeneration to insult, the nervous system is very sensitive to damage, unlike other organs. Studies on the uptake of metallic nanoparticles have demonstrated that they can pass the blood–brain barrier and cause toxic effects on the cells of the nervous system, including neurons, glia, and microglia (review [6,7]). Inhalation of nanoparticles through the nose can lead to entry into the brain via migration along the olfactory bulb [8,9,10]. Another concern is that nanoparticles that do not cross the blood–brain barrier under adult conditions may be neurotoxic because, in earlier embryonic stages, the barrier is not yet fully developed. 

While the design of nanoparticles underscored by green nanotechnology is meant to minimize their toxicity, the use of plant extract may have unexpected impacts in addition to the effect of pure nanoparticles on their own. Thus, there is an urge for testing their potential toxicity, employing effective test strategies [11]. Classical methods for chemical risk assessment are animal studies to evaluate possible adverse effects on human health. However, studies in experimental animals are time consuming, expensive, require large numbers of animals, and may be of limited biological relevance [12,13], in addition to presenting animal ethical concerns. One of the few vertebrate alternatives are zebrafish embryos, which are commonly used for assaying nanoparticle toxicity (review [14]). However, alternative approaches using cell-based tests are often less resource-intensive and may faster yield results than traditional animal toxicity methods. 

Up to now, the developmental neurotoxic (DNT) potential of nanoparticles for human brain development has rarely been addressed. In a study on human embryonic stem cell-derived neuronal precursor cells, Hoelting et al. reported the DNT potential of polyethylene nanoparticles [15]. To explore potentially adverse effects on human nerve cells, here we utilize a predictive in-vitro test system for developmental neurotoxicity [16,17]. This system is based on a well-characterized teratocarcinoma cell line (Ntera-2, NT-2) derived from a human testicular cancer [18,19]. Differentiated NT-2 neurons are clonally derived, but express the characteristics of primary human neuron cultures [20]. Thus, they are experimentally advantageous cells to characterize neurotoxic and neuroprotective compounds [21,22,23,24,25]. Using a rapid differentiation step in cell aggregates [26], we induce this cell line to differentiate into fully functional post-mitotic neurons by exposure to retinoic acid [18,19]. The multiple phenotypes of these neurons have already been determined by immunolabelling for classical neurotransmitters [27]. Electrophysiological recordings have uncovered synaptic connections among the cultured NT-2 neurons [28] and a fluorescence assay showed Botulinum toxin A-sensitive neurotransmitter vesicle release [29]. 

A special feature of brain development is the extensive cell migration of newly born neurons before they start to extend their neurites [30,31]. We have compared the in-vitro motility of differentiating NT-2 neurons [32] to the motility of neural progenitor cells obtained from human fetal tissue [33]. Both developing neuronal cell types respond to exogenous application of nitric oxide (NO), with the synthesis of cGMP. Moreover, they show a similar facilitation of motility, demonstrating a common expression of NO/cGMP signal transduction as a positive regulator of cell migration [33]. 

The aim of this study was to characterize the cytotoxic effects of CuS nanoparticles on the viability of cultured NT-2 precursor cells and developing neurons. To quantify developmental neurotoxic effects, we measured, using concentration–response curves, the migration and neurite outgrowth of differentiating NT-2 neurons in comparison to their cell viability. Thus, our nanotoxicological analysis initially focuses on two important steps of early nervous system development.

## 2. Results

### 2.1. General Cytotoxicity

**Figure 1:**
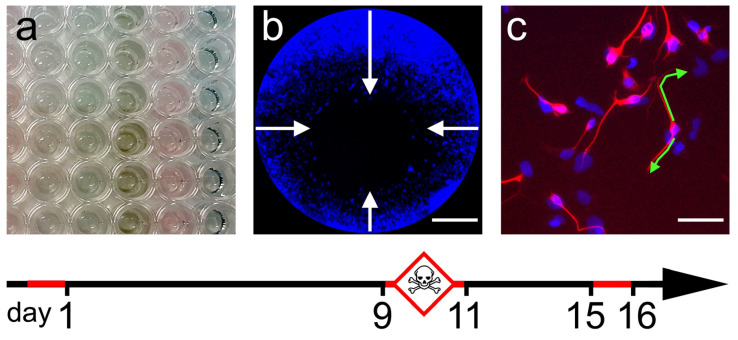
Three endpoints assessed on NT-2 cultures in vitro. (**a**) General cytotoxicity was assessed by measuring the fluorescence of resazurin converted into resorufin by mitochondrial activity of undifferentiated NT-2 cells. (**b**) NT-2 precursor cell migration was measured using the Oris cell migration assay, which creates cell culture monolayers with a circular hole. During 2 days in culture, cells migrated a distance of ~400 µm on average into this hole. The white arrows indicate direction of cell migration. (**c**) Neurite growth was assessed by cultivating dissociated NT-2 cultures after 2 weeks of exposure to retinoic acid (containing ~20–40% neurons) and measuring ß-tubulin type III labelled neurites (red) after 24 h. Green lines: example measurements of two neurites, 46 µm and 76 µm long. Scale bars: 400 µm (B) and 50 µm (C). (modified after [17], http://creativecommons.org/licenses/by/4.0/ accessed on 14 April 2024).

**Figure 2:**
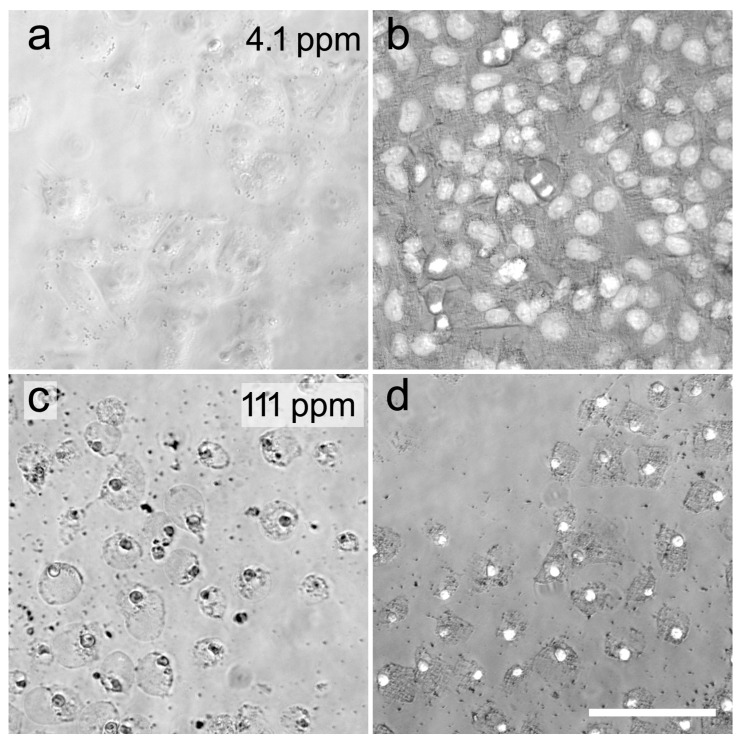
Example cultures of undifferentiated NT-2 cells treated for 18 h with 4.1 ppm (**a**,**b**) or 111 ppm nanoparticles (**c**,**d**). Phase contrast images in b and d, combined with DAPI fluorescence of nuclei. Scale bar 100 µm.

**Figure 3:**
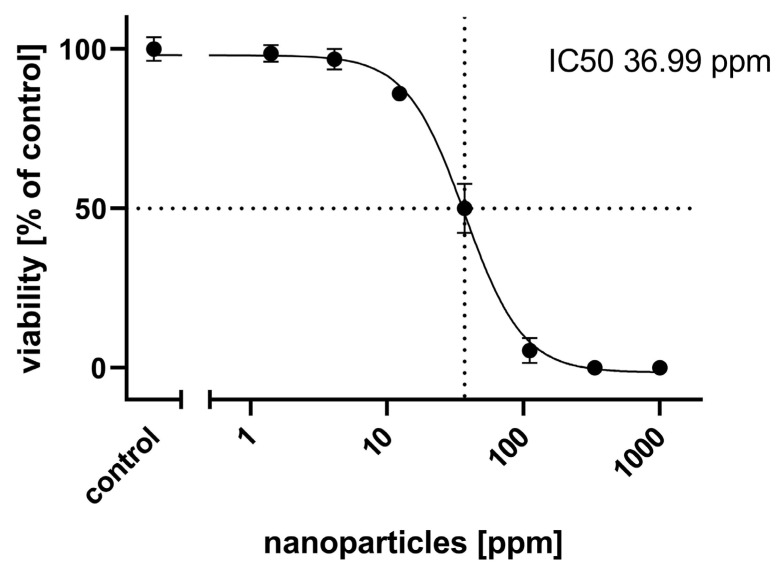
General cytotoxicity concentration–response curve: each value is the average ± S.E.M. of five independent experiments normalized to the control. The IC50 (dotted lines) was 36.99 ppm.

When NT-2 cells were seeded in 96-well plates at 10,000 cells/well in normal media without retinoic acid, they adhered to the plastic surface within 1 h and proliferated to 90% confluency within 24 h. Dispersed CuS nanoparticles caused a greenish color of the cell culture media (Figure 1a) at high concentrations. At a concentration of 4.1 ppm, cells appeared viable and had grown to 90% confluency (Figure 2a). Nuclear labelling with DAPI revealed multiple mitoses, indicating unimpeded proliferation (Figure 2b). At 111 ppm, cells had not proliferated and appeared dead (Figure 2c), and nuclear labelling revealed condensed necrotic nuclei and no mitoses (Figure 2d). When testing over a wide range of nanoparticle concentrations from 1 ppm to 1000 ppm, a concentration-dependent reduction of viability was observed, with an IC50 of 36.99 ppm (Figure 3).

### 2.2. Cell Migration Assay

**Figure 4:**
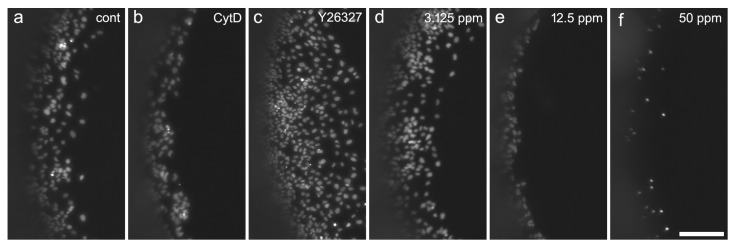
Example of NT-2 cells after 9 days of differentiation and 44 h exposed to test compounds; nuclei labelled with DAPI. Cells were exposed to (**a**) the cell culture media only (cont), (**b**) 100 µM cytochalasin D (Cyt D), (**c**) 50 µM Y-27632, (**d**) 3.125 ppm nanoparticles, (**e**) 12.5 ppm nanoparticles, and (**f**) 50 ppm nanoparticles. Scale bar 200 µm.

NT-2 cells are well suited for the quantification of the specific DNT potential of toxic compounds on a neuronal precursor [16,17]. When cells were seeded in 96-well plates with circular silicon stoppers, cells migrated into the free space after removal of the stopper (Figure 1b and Figure 4). Within 44 h, the fastest cells migrated a distance of more than 200 µm under control conditions in the cell culture media only (Figure 4a). Migration could be inhibited by cytoskeletal inhibitors, such as cytochalasin D [16], and enhanced by the ROCK inhibitor, Y-27632 (Figure 4c), allowing us to monitor both the increase and reduction in cell migration upon treatment with chemicals. 

**Figure 5:**
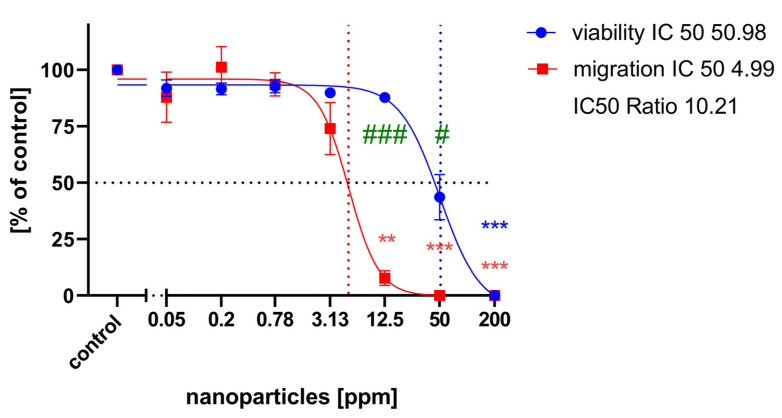
Migration assay concentration-response curves: each value is the average ± S.E.M. of three independent experiments normalized to the control. Blue circles: general cytotoxicity (resazurin); red circles: migration distance. Asterisks indicate significant differences (** *p* < 0.01, *** *p* < 0.005) from controls; (# *p*< 0.05, ### *p* < 0.005) indicate significant differences between viability and migration at the specified concentration. IC50 values are indicated by dotted lines.

When treated with 12.5 ppm nanoparticles, migration was strongly reduced (Figure 4e), whereas migration appeared unaffected at 3.125 ppm (Figure 4d). At 50 ppm, no migration was detectable, and only a few condensed nuclei remained (Figure 4f). Migration was reduced in a concentration-dependent manner with an IC50 of 4.99, whereas general viability was reduced at a higher concentration with an IC50 of 50.98 (Figure 5). A significant reduction in migration could be observed at 12.5 ppm, where cell viability was not significantly affected. The IC50 ratio between general cytotoxicity and inhibition of cell migration was 10.21.

### 2.3. Neurite Outgrowth Assay

**Figure 6:**
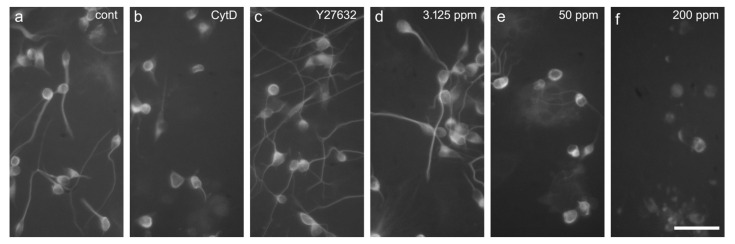
Neurite outgrowth: example of NT-2 neurons after 14 days of differentiation and 24 h exposed to test compounds, immunolabelled against ß-tubulin Type III. Cells were exposed to (**a**) the cell culture media only (cont), (**b**) 100 µM cytochalasin D (Cyt D), (**c**) 50 µM Y-27632, (**d**) 3.125 ppm nanoparticles, (**e**) 50 ppm nanoparticles, and (**f**) 200 ppm nanoparticles. Scale bar 50 µm.

As established before [17,23], NT-2 cells treated for 2 weeks with 10 µM retinoic acid in non-adherent dishes and subsequently plated on poly-D-lysine, contained ~20% post-mitotic, ß-tubulin type III-positive neurons, of which a large portion readily grew neurites within 24 h (Figure 1c and Figure 6a). Neurite outgrowth could be enhanced by application of ROCK inhibitors, like Y-27632 ([17], Figure 6c), and reduced by the cytoskeletal inhibitor, cytochalasin D (Figure 6b), again allowing us to observe changes to neurite outgrowth in both directions. 

**Figure 7:**
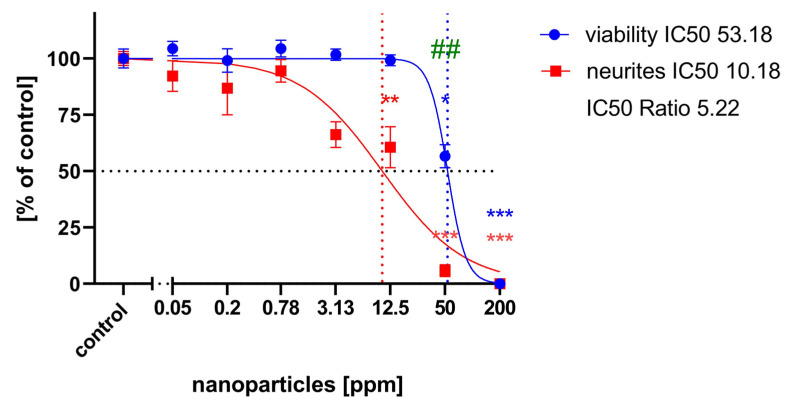
Neurite outgrowth concentration-response curves: each value is the average ± S.E.M. of three independent experiments normalized to the control. Blue circles: general cytotoxicity (resazurin); red circles: neurite length/cell. Asterisks (*) indicate significant differences (* *p* < 0.05, ** *p* < 0.01, *** *p* < 0.005) from controls; (## *p* < 0.01) indicate significant differences between viability and neurite outgrowth at that concentration. IC50 values are indicated by dotted lines.

When treated with low concentrations of CuS nanoparticles (3.125 ppm), neurite outgrowth appeared unaffected (Figure 6d), whereas high concentrations (12.5 ppm) strongly inhibited outgrowth (Figure 6e). At even higher concentrations, no neurite growth was observed, and many cells lost integrity and displayed lower ß-tubulin III immunofluorescence (Figure 6f). The application of nanoparticles over a large range of concentrations (0.2 to 200 ppm) revealed a concentration-dependent inhibition of neurite outgrowth, with an IC50 of 10.18 ppm (Figure 7). General cell viability was also significantly reduced compared to the control at higher nanoparticle concentrations, with an IC50 of 53.18 ppm. Comparing the IC50 values for neurite outgrowth and general cytotoxicity resulted in a ratio of 5.22.

## 3. Discussion

Developmental neurotoxic compounds are substances that exert adverse impacts on the normal development of the human nervous system. To examine the DNT potential of our phyto-engineered CuS nanoparticles, in this study we used a predictive in-vitro test system [16]. This test assay contained differentiating NT-2 human neurons, which acquired a post-mitotic stage, resulting in immunofluorescence staining for the neuron-specific cytoskeletal marker ß-tubulin type III (Figure 1c) and other markers [27]. Similar to fetal development, the differentiating precursors migrated in vitro (Figure 1b) and eventually showed neurite outgrowth (Figure 1c). After nanoparticle incubation at day 9 in vitro, we determined endpoints for cell migration and neurite outgrowth, as read out of the assay. 

To visualize how increasing concentrations of CuS nanoparticles affected the proliferation of NT-2 precursor cells, we performed nuclear DAPI staining (Figure 2). As shown by chromosomal labelling, the cells tolerated a low concentration of nanoparticle exposure. Both intact nuclei and mitotic stages were visible at a concentration of 4.1 ppm (Figure 2b). However, at 111 ppm, the majority of cells appeared dead with rounded morphology and nuclear shrinkage, indicating necrotic pyknosis (Figure 2c,d). 

In a first range-finding approach, the resazurin assay determined an IC50 of 36.99 ppm (Figure 3) for general cytotoxicity. This viability value corresponds well to another study about the safety of CuS nanoparticles, which were produced by microwave-assisted synthesis [34]. Using a human skin cancer cell line as the in-vitro test system, these authors determined a general cytotoxicity of 12.49 ppm at IC50. However, to the best of our knowledge, the issue of a potential developmental neurotoxicity of CuS nanoparticles has so far not been investigated. 

As shown in Figure 5, the concentration-response curve for cell migration is shifted to the lower concentrations compared to the curve for general cytotoxicity. The IC50 ratio between general cytotoxicity and inhibition of cell migration was 10.21, which is clearly above the threshold of 2.30, as calculated from the IC50 ratios of DNT negative compounds [17]. This strongly indicates a specific DNT effect of copper sulfide nanoparticles on neuronal precursor cell migration. The quantification of neurite length also showed a blocking of neurite outgrowth at lower concentrations than the cell viability curve. Comparing the IC50 values for neurite outgrowth and general cytotoxicity resulted in a ratio of 5.22. Krug et al. [35] assumed IC50 ratios above a threshold value of 4 as indicative for DNT on neurite outgrowth of LUHMES cells, defined by the average ratio + 3× standard deviation of unspecific test compounds. The threshold IC50 ratio for NT-2 neurite outgrowth is 2.02 [17], which clearly exceeded for copper sulfide nanoparticles in our experiments, confirming their DNT potential. 

DNT assays require endpoint-specific controls with known modes of actions [36]. Cytochalasin D is a cell-permeable blocker of actin filament polymerization, while the drug Y-27632 targets the Rho kinase (ROCK), promoting an increase in neurite length of NT-2 neurons [23]. Indeed, cell migration could be inhibited by the cytoskeletal inhibitors cytochalasin D ([16], see Figure 4b, and enhanced by the ROCK inhibitor, Y-27634 (Figure 4c)), allowing us to monitor both the increase and reduction in cellular motility upon treatment with chemicals of known actions.

Nanoparticles are explored in biomedical applications, such as biosensors, nanocarriers, and in the ablation of cancer cells [37]. Easy removal of smaller sized nanoparticles than ours through the kidneys implies a low toxicity for the organism [38]. However, despite the environmentally friendly phyto-engineered synthesis of CuS nanoparticles [4], the combined data of our study provide considerable safety concerns, because there is clear evidence for a developmental toxic classification on tests with human nerve cells. So far, we have not addressed the cellular mechanisms responsible for the developmental neurotoxic effects of nanoparticles. For example, nasal exposure of CuO nanoparticles in mice has led to severe lesions in the brain, which could be caused by oxidative stress in nerve cells [39]. The generation of free radicals and induction of oxidative stress are likely to be common mechanisms of neurotoxicity caused by metal nanoparticles [1,6]. Our assay would allow for testing this assumption. Past experiments showed that an anti-oxidative substance could counteract the inhibitory effects of the insecticide Fipronil on cell migration [17], indicating the involvement of oxidative stress as a mechanism. 

Another issue relates to the question of whether CuS nanoparticles might cross the blood–brain barrier. To resolve this issue, animal experiments, preferentially with rodents, are required [40]. However, there are also alternative methods available, which can serve as a model for the vertebrate blood–brain barrier. Insects also protect their nervous system with a blood–brain barrier, which is often termed the hemolymph–brain barrier. This barrier comprises perineural and subperineural glia, which are linked by pleated septate junctions, equivalent to tight junctions [41]. To assess brain uptake of drugs in vertebrates, the brain of locusts has been advocated as a novel ex-vivo model [42]. The tiny insect brain, with a relatively large surface, is incubated with test compounds and uptake is determined by liquid chromatography–mass spectrometry. The uptake of 25 known drugs over the hemolymph–brain barrier resulted in a linear correlation with in-situ perfusion data from vertebrates including P-glycoprotein (PgP) transporter substrates [42]. A kinetic analysis can separate barrier permeability, drug metabolism, and efflux [43]. Conserved mechanisms between efflux transporters in insects and mammals are also supported by the transcriptomic identification of a human P-glycoprotein in locusts [44]. Since we have also designed embryonic locust models for evaluating developmental toxicity [45,46], it is now a realistic goal to elucidate the neurotoxic mechanisms of CuS nanoparticles with new approach methodologies (NAMs). Such experiments could be the key to understanding how CuS nanoparticles cross the blood–brain barrier and how they elicit adverse effects on the developing and mature nervous system. A further mechanism of nanoparticle DNT could be adverse effects on microglia cells, the immune cells of the nervous system. Luther et al. [47] demonstrated that iron oxide nanoparticles are readily taken up by microglia mediated by macropinocytosis and clathrin-mediated endocytosis, which direct the accumulated particles into the lysosomal compartment [47].

The awareness for possible adverse effects of nanoparticles on human health is currently strengthening [1]. Since nanoparticles are increasingly used for medical applications, such as tumor therapy [38] or drug delivery for the treatment of eye diseases [48,49], it will be important to carefully explore potential adverse non-target effects. Furthermore, nanoparticles are discussed in pest control [50]. Based on the toxicity of green-synthesized copper nanoparticles using Spodoptera larvae, Barathi et al. [51] derived a rather high larvicidal and antimicrobial activity. Another study evaluated the larvicidal potential of Cu nanoparticles against mosquito larvae [52]. This possible usefulness of nanoparticles as insecticides implies both direct occupational exposure to manufacturers and farmers, and considerable release into the environment and accumulation in field crops, leading to nanoparticle exposure to consumers. To our knowledge, there are no data available about the environmental concentrations of this engineered type of nanomaterial. Environmental concentrations would clearly depend on the source of measurements, such as air, surface water, ground water, soil, sediment, biomaterials etc. To provide an example, analytical measurements for very toxic ZnO nanoparticles in canal water are within the 1–10 µg/L range (0.001–0.01 ppm) [53,54]. Assuming a similar level of pollution, one can speculate that expectable general environmental concentrations of CuS nanoparticles would be several degrees of magnitude below the IC50 values in our neurotoxicological study. However, occupational exposure levels during the production or application of NPs might well fall into the toxicological relevant range. Various potential biomedical applications for CuS nanoparticles have been summarized by Ain and colleagues [38]. Nanoparticle concentrations vary depending on the mode of action and application, but can be estimated to be in or close to the toxicologically relevant ppm range. In any case, the determination of toxic potential is a crucial demand for assessing the safety of these promising materials.

## 4. Materials and Methods

### 4.1. Chemicals

All chemicals were obtained from Merck, Darmstadt, Germany, unless stated otherwise. Cytochalasin D was dissolved in dimethyl-sulfoxide (DMSO) as a stock solution, which was further diluted in the cell culture media, resulting in a final concentration of 0.1% DMSO in culture media. In previous experiments, DMSO concentrations up to 1% had no observable adverse effects on our cell cultures [17]. Y-27632 ((1R,4r)-4-[(R)-1-Aminoethyl]-N-(pyridin-4-yl)cyclohexancarboxamide) was dissolved directly in the cell culture media. 

### 4.2. Nanoparticles

A detailed description of the generation and characterization of copper sulfide nanoparticles is given elsewhere [4]. In brief, CuS nanoparticles of spherical and semi-spherical shape and an average size of 77.89 nm were synthesized using avocado seed extract and green chemistry, as described in [4]. Copper sulfide nanoparticles were autoclaved at 120 °C for 1 h in test tubes, suspended in the cell culture media at a concentration of 1000 ppm, vortexed for 30 s, and sonicated for 3 h in an ultrasound bath (Transsonic T700/H, Elma, Singen, Germany), before serial dilution and immediate application to cell cultures.

### 4.3. Cell Culture

The human Ntera2/D1 cell line (NT-2, RRID: CVCL_3407) was obtained from the American Type Culture Collection, VA, USA. Cells were maintained and cultivated in DMEM/F12 culture medium (Invitrogen, Darmstadt, Germany), supplemented with 10% fetal bovine serum (Invitrogen) and 1% penicillin/streptomycin (Invitrogen) in an atmosphere of 5% CO2 at 37 °C. Using a differentiation protocol in free-floating aggregates [26], pure post-mitotic neurons could be generated within 28 days, with the aid of the morphogen, retinoic acid (RA, 10 µM). General cytotoxicity, neuronal precursor cell migration, and neurite outgrowth were measured in 96-well plates on days 1, 9, or 11, respectively (Figure 1). Each plate contained a dilution series of seven concentrations with six technical replicates of the same concentration per experiment, and each experiment was performed three times on different passages of NT-2 precursor cells.

### 4.4. General Cytotoxicity

Undifferentiated NT-2 precursor cells stored in the cell culture media with 10% DMSO at −176 °C were thawed and grown to 90% confluency in T175 Flasks (Greiner, Hamburg, Germany). Cells were trypsinized, resuspended in DMEM/F12 without RA, and seeded at 10,000 cells/well in 200 µL media into uncoated 96-well-plates (Corning Costar, Kaiserslautern, Germany). After adhesion for 1 h, cells were exposed to seven different concentrations of copper sulfide nanoparticles in DMEM/F12 (six technical replicates per concentration, Figure 1a). After 24 h incubation, a resazurin reduction assay (Invitrogen) was performed for 2 h, before the cells were fixed in 4% paraformaldehyde in PBS (PFA) for 15 min. Cultures were incubated in DAPI (0.1 µg/mL) for 5 min to label nuclei. Cells were examined and examples photographed on a Zeiss Axiovert 200 inverted microscope, equipped with a Colibri LED light source and a Zeiss Axiocam50 mono digital camera and Zeiss ZENlite 2.6 blue edition software. 

### 4.5. Cell Migration Assay

As established before [16,17], NT-2 precursor cells were seeded in 96 mm bacteriological grade Petri dishes (Greiner, Hamburg, Germany) at a density of 4 × 106–5 × 106 cells per dish. Within 24 h, cells formed free-floating spherical aggregates of 300–800 µm diameter. On the first day, 10 mL of the culture medium was added to each Petri dish. On the next days, 10 μM retinoic acid was added and medium was changed every 2–3 days by transferring the cell suspension to centrifuge tubes and centrifuging at 200× *g* for 7 min. After 9 days in culture, aggregates were gently resuspended using a Pasteur pipette and seeded at 60,000 cells per well into black 96-well plates with a flat transparent bottom (Nunc). Plates were lined with poly-D-lysine and laminin (10 µg/mL each), equipped with silicone stoppers from the Oris Cell Migration Assay (AMS Biotechnology, Abingdon, UK). Cells were allowed to adhere overnight. On the next day, stoppers were pulled, leaving a monolayer of cells with a circular hole of 2 mm diameter. After one washing step to remove non-adherent cells, cultures were exposed to seven different concentrations of copper sulfide nanoparticles in DMEM/F12/RA (six technical replicates per concentration) for 44 h, followed by resazurin reduction viability assay (2 h). After one wash in PBS, cells were fixed for 15 min in PFA, washed twice in PBS-T and stained with DAPI (0.5 µg/mL) for 5 min, followed by two washes in PBS. A black 96-hole plastic mask leaving a central circular area of 2 mm diameter for each well was clipped to the bottom of the plate. This allowed viewing only those cells that had migrated into the free areas left by the silicone stoppers during seeding (Figure 1b). Migration was quantified by taking two photographs of each well and measuring the distance of the migration front from the black margin of the mask using ImageJ (http://imagej.nih.gov/ij accessed on 1 February 2024). Occasional asymmetries caused by slight inaccuracies of silicone stopper placement were compensated by averaging measurements in four quadrants per well. As a null-migration reference, six wells per plate were incubated with 100 nM cytochalasin D, which completely inhibits cell motility without affecting viability within 44 h [16]. Average null-migration values were subtracted from the migrated distance before evaluation. As an endpoint-specific control, cells in six wells were subjected to the Rho-kinase inhibitor, Y-27632 (50 µM), which increases precursor cell migration to more than 125% without affecting general viability. Cell cultures that did not meet these criteria were discarded.

### 4.6. Neurite Outgrowth Assay

Similar to our previous studies [17,23], experiments were performed on NT-2 cells treated for 2 weeks with RA from passage 27 to 35. Cells were gently dissociated using a glass Pasteur pipette and seeded into two identical poly-D-lysine (10 µg/mL) coated 96-well-plates (Corning Costar, Kaiserslautern, Germany) at a density of 10,000 cells per well. Cultures were exposed to seven different concentrations of CuS nanoparticles in DMEM/F12 (two technical replicates per concentration) for 24 h. One plate was immediately fixed in PFA for 15 min, while a resazurin reduction assay (2 h) was performed on the other plate. This was necessary, because even completely inhibited neurites immediately started growing in the resazurin assay. Fixed cells were washed in PBS containing 0.1% Triton X100 (PBS-T), and immunolabelled for ß-tubulin type III (monoclonal anti ßtubIII, 1:20,000) using a red fluorescent secondary antibody (goat anti mouse-alexaFluor568, Invitrogen, Germany, 1:250). Two photographs per well at 40× magnification were taken. Lengths of all neurites in the field of view were measured in ImageJ using the ‘segmented line’ tool (Figure 1c) and divided by the number of ß-tubulin III-positive cells. As a null outgrowth reference, cells were subjected to cytochalasin D (100 nM), which reduces outgrowth to less than 25% compared to the control (not to 0%, because some neurons displayed residual neurite stumps that remained after cell dissociation). Average null-outgrowth values were subtracted from neurite lengths before evaluation. As an endpoint-specific control, cells in two wells were subjected to the rho-kinase inhibitor, Y-27632 (50 µM), which increases neurite outgrowth to more than 125% without affecting general viability. Cell cultures that did not meet these criteria were discarded.

### 4.7. Statistics and Prediction Model

Concentration-response relationships were displayed as the mean ± S.E.M. of three independent experiments using various passages of NT-2-cells, normalized to untreated controls. To determine IC50 values, 4-parameter logistic curves were fitted to the data using GraphPad Prism 9.0.0. Differences between individual values were evaluated by one-way ANOVA, followed by Dunnett’s test for multiple comparisons. 

Statistical differences between general cytotoxicity and DNT specific endpoints can indicate the DNT potential of a test compound. A more reliable prediction model should include the variation in responses of the test system to both DNT-specific and non-specific compounds [34,35], assuming IC50 ratios above a threshold value of 4 as indicative for DNT on neurite outgrowth of LUHMES cells, defined by the average ratio + 3× standard deviation of unspecific test compounds. We applied the same procedure for neurite outgrowth and precursor cell migration of NT-2 neurons using five different unspecific compounds and arrived at a threshold value of 2.02 for neurite outgrowth and 2.30 for precursor cell migration, respectively [17].

## Data Availability

The raw data supporting the conclusions of this article will be made available by the authors on request.
